# Proteomic and Transcriptomic Profiling Identifies Early Developmentally Regulated Proteins in *Dictyostelium Discoideum*

**DOI:** 10.3390/cells8101187

**Published:** 2019-10-01

**Authors:** Óscar González-Velasco, Javier De Las Rivas, Jesus Lacal

**Affiliations:** 1 Bioinformatics and Functional Genomics Research Group. Cancer Research Center (CIC-IBMCC, CSIC/USAL/IBSAL), 37007 Salamanca, Spain; oscargv@usal.es (Ó.G.-V.); jrivas@usal.es (J.D.L.R.); 2 Department of Microbiology and Genetics, Faculty of Biology, University of Salamanca, 37007 Salamanca, Spain

**Keywords:** bioinformatics, chemotaxis, early differentiation, cAMP, *Dictyostelium*

## Abstract

Cyclic AMP acts as a secondary messenger involving different cellular functions in eukaryotes. Here, proteomic and transcriptomic profiling has been combined to identify novel early developmentally regulated proteins in eukaryote cells. These proteomic and transcriptomic experiments were performed in Dictyostelium discoideum given the unique advantages that this organism offers as a eukaryotic model for cell motility and as a nonmammalian model of human disease. By comparing whole-cell proteome analysis of developed (cAMP-pulsed) wild-type AX2 cells and an independent transcriptomic analysis of developed wild-type AX4 cells, our results show that up to 70% of the identified proteins overlap in the two independent studies. Among them, we have found 26 proteins previously related to cAMP signaling and identified 110 novel proteins involved in calcium signaling, adhesion, actin cytoskeleton, the ubiquitin-proteasome pathway, metabolism, and proteins that previously lacked any annotation. Our study validates previous findings, mostly for the canonical cAMP-pathway, and also generates further insight into the complexity of the transcriptomic changes during early development. This article also compares proteomic data between parental and cells lacking glkA, a GSK-3 kinase implicated in substrate adhesion and chemotaxis in Dictyostelium. This analysis reveals a set of proteins that show differences in expression in the two strains as well as overlapping protein level changes independent of GlkA.

## 1. Introduction

The soil-dwelling social amoeba *Dictyostelium discoideum* is an excellent model organism for the study of directed cell migration since *Dictyostelium* cells show robust chemotactic responses to the chemoattractant cAMP [1,2,3,4]. Chemotaxis is a dynamic process that involves directional sensing, cell polarity, and eventually directed cell motility. Cell migration is fundamental to establishing and maintaining the proper organization of multicellular organisms, from large-scale migrations of epithelial sheets during gastrulation, to the movement of individual cells during development of the nervous system [5,6,7]. In an adult organism, cell migration is essential for proper immune response [8], wound repair [9], and tissue homeostasis [10], while aberrant cell migration is found in various pathologies [7].

In *D. discoideum,* actin polymerization in pseudopods at the leading edge of the cell, is synchronized with contractile forces generated by myosin motor proteins at the rear [11,12]. A directional sensing system biases pseudopodia formation towards the source of the chemoattractant, and thus orients cell movement along the cAMP gradient. This process is achieved by the ability to signal each other by secreting cAMP. The molecular mechanisms underlying chemotaxis, such as actin polymerization, intracellular signaling, and cell migration, are highly conserved among eukaryotes. Indeed, previous studies have demonstrated that many components involved in chemotaxis, are functionally conserved between human neutrophils and *Dictyostelium* amoebae [13,14,15].

*Dictyostelium* cells communicate through the production of diffusible signaling molecules that accumulate, and once a critical concentration has been reached, regulate transcription of a number of target genes. As long as nutrients are present, *Dictyostelium* cells grow and multiply as unicellular amoeba (vegetative growth). During vegetative growth AprA, DfaD and the pre-starvation factor PSF are the signals used for quorum sensing that regulate cell proliferation [16,17,18]. The development of *Dictyostelium* is triggered by starvation. The signal that initiates development is the lack of available nutrients and triggers dramatic changes in transcription that adapt the cells to a period of starvation. When cells begin to starve, triggered by nitrogen limitation, they enter a developmental cycle and signal other cells by secreting an array of factors such as the glycoprotein conditioned medium factor (CMF) [18,19]. During starvation CMF signaling at 2 h of development activates CMF receptor, inhibiting PldB activity and thus increasing cAMP signaling [20]. Within a few h, the cells begin to accumulate and secrete cAMP, which is used both as a chemoattractant and an intercellular signal. During its differentiation, ~100,000 cells migrate toward aggregation centers that release the chemoattractant cAMP and form multicellular structures [21,22]. At 4 h of development cAMP pulses start, and differentiating cells secrete cAMP every 6 min, and waves of extracellular cAMP reinforce the expression of the cAMP receptors and other signaling molecules that are required to respond to cAMP. Most of the cAMP is secreted to the extracellular buffer where it can diffuse to adjacent cells. The level and shape of the cAMP waves that are relayed outwards from the center of an aggregate are controlled by the activity of the extracellular cAMP phosphodiesterase, PDE1 [23,24,25]. Therefore, extracellular cAMP is rapidly degraded and must be continuously replenished. Nearby cells will entrain each other such that they all synchronously produce cAMP in waves [26,27]. These cAMP pulses are used both for chemotactic directionality and induction of the early pulse dependent genes [28]. These pulse-dependent genes include those coding for the aggregation stage adenylyl cyclase, ACA, the cell adhesion proteins, CsaA (gp80) and TgrC1, and five calcium-binding proteins. There are other genes which expression is mediated by the rise in internal cAMP, like the ACA-dependent genes [29]. The communal aspects of extracellular cAMP to which all cells respond resynchronizes the transcriptional profiles of cells after 5 to 8 h of development. Therefore, differentiation normally takes several h, and the chemotactic ability peaks at 5–6 h after starvation [30,31]. Around this time, cells establish an increased cell polarity due to downregulation of basal cytoskeletal activity, and become highly sensitive to chemoattractant stimulation. Once the cells have formed the aggregates, they continue to communicate with each other using a series of signals. At 9 h TgrB/C and DIF-1 at 12 h approximately, will lead to the slug stage [32]. During culmination, the resulting transcriptional changes at approximately 20 h of development, caused by MPBD, SDF-1, steroid, GABA, glutamate, SDF-2, cytokinin and cyclic diGMP, in that particular order, will ultimately lead to differentiation into either dormant spores or vacuolized stalk cells [29,33,34,35]. Development finishes with the formation of fruiting bodies at approximately 24 h.

The genome of *D. discoideum* was among the first eukaryotes to be sequenced [36]. The technological and experimental advances allowed the study of the developmental transcriptome, from the early days of gene expression microarrays to modern whole genome sequencing of mutants and proteomics [37,38]. The genome-wide expression and proteomics analyses with wild-type and mutant strains, have characterized a large number of developmentally regulated genes, including but not limited to genes of the canonical cAMP-pathway such as the cAMP receptor (*carA*), the Gα subunit that couples the receptor to adenylyl cyclase (*gpaB*), the activatable adenylyl cyclase (*acaA*) and the secreted and internal cAMP phosphodiesterases (*pde1* and *regA*) [39,40,41,42,43].

Using genome-wide transcriptional analysis it was shown that the largest transition in the transcription pattern occurs at 6 h after starvation, coincident with the transition from unicellular to multicellular organism. This process has been reported to involve changes in expression of more than 25% of the genes in the genome, using a DNA microarray that consists of hybridization targets for about 75% of all *D. discoideum* genes [1]. Similarly, in order to characterize a large proportion of the genome, it was arrayed 5655 targets from cDNAs provided by the Japanese EST Project, together with 690 targets previously chosen from specific genes, recognizing 172 genes highly expressed during development of *D. discoideum*. In particular, when developed in shaken suspension, 125 of these genes were expressed when cells were treated with cAMP pulses at 6-min intervals, between 2 and 6 h of development, followed by high levels of exogenous cAMP [28]. Just a few years later it was suggested that gene function can be inferred from co-expression by including a clustering analysis of genes involved in various aspects of chemotaxis [44]. More recently, proteomic iTRAQ analysis showed that there are quantitative differences in the pattern of protein expression depending on the growth conditions in AX2 wild-type cells and *glkA* null cells that were pulsed with cAMP for 5 h 30 min [45]. GlkA is a GSK family protein kinase, that is required for the proper regulation of growth, chemotaxis and multicellular development. Indeed, GlkA has overlapping functions with *Dictyostelium* GSK3 in controlling early development, in part through regulation of MyoII, Rap1 and RasG [45].

In humans, cAMP has been reported to have both intracellular and extracellular functions. cAMP is secreted from multiple tissues including kidney [46], liver [47] and adipose tissue [48] and cell types such as hepatocytes [49], glomerular epithelial cells [50], renal proximal tubule cells [51], and adipocytes [52]. Glucagon not only stimulates intracellular cAMP accumulation in the liver, but also induces a significant release of liver-borne cAMP into the blood [53,54]. Overall, cAMP contribution from normal to metabolic dysfunction still requires further investigation.

In this work, we have used bioinformatics to combine previous transcriptomic [55] and proteomic [45] data in which we and others compared the effect of cAMP stimulation in gene and protein levels, respectively. We have identified a proteomic and transcriptomic signature of early developmentally regulated proteins, including 26 proteins previously related to cAMP signaling and 110 novel proteins involved in calcium signaling, adhesion, actin cytoskeleton, the ubiquitin-proteasome pathway, metabolism, and proteins that previously lacked any annotation. We have analyzed all these proteins individually, and assigned them with a biological role based on their biological annotations, orthologs information, and previous published findings. We have also compared the proteome of wild-type AX2 and *glkA* null (*glkA*^−^) cells. GlkA is a GSK family protein kinase, that is required for the proper regulation of growth, chemotaxis, and multicellular development [45]. With the comparison of AX2 and *glkA*^−^ proteomes we found a set of proteins which levels depend on GlkA. To our knowledge, this is the first report combining whole *D. discoideum* genome transcriptomics and proteomics. Our analyses have validated previous findings, mostly for the canonical cAMP-pathway, and also generate further insight into the complexity of the cAMP-signaling response.

## 2. Materials and Methods

### 2.1. Identification of Protein Level Changes in Response to cAMP Pulsing Using Proteomics in Dictyostelium

In order to identify quantitative differences in the proteome of developed (cAMP-pulsed) and vegetative (non-cAMP pulsed) *Dictyostelium* cells (wild-type AX2 and *glkA* null cells) we analyzed the raw data from a previous proteomic iTRAQ assay [45]. First, out of the total number of 5149 proteins in the MS/MS data set coming from 24 biological samples, we filtered those identified proteins with iTRAQ values over zero in at least 14 out of the 24 samples. This cut off yielded a total of 2973 proteins. We then performed a log2 transformation of the filtered proteins, and used the statistical programing language R and the Bioconductor package limma. Additionally, pair-wise differential expression analysis was performed between vegetative and developmental (cAMP-pulsed) conditions. Last, we selected proteins within the top 500 absolute fold-change values given by limma, which also conserved the negativeness or positiveness among tests on the analysis (concordant fold change values). The above protocol yielded 199 proteins in AX2 cells and 197 proteins in *glkA* null cells.

In a different analysis, we look at the MS/MS data set to identify GlkA-dependent protein level changes. To identify these potential candidates, those proteins that were not detected at all in *glkA* null samples but were found in AX2 samples were selected for further analysis. Similarly, proteins with zero iTRAQ values in all AX2 samples but over zero in all *glkA* null samples were also selected. To compare if the presence/absence of these proteins in AX2 using proteomics happened in transcriptomics, our proteomics data was compared to a previous transcriptomics assay [55] (GEO dataset: GSE61914) that was performed under similar experimental conditions. First, we established a baseline threshold as a reference measurement to determine whether a gene was expressed or not: This value was set as the basal expression of the global transcriptome, and was calculated as the mean expression of all genes at 0 h, 4 h, 5 h, and 6 h. Finally, the mean expression of the targeted zero signal genes was calculated at 0 h, 4 h, 5 h, and 6 h, and contrasted to the basal global expression.

### 2.2. Functional Annotation Clustering of Identified Proteins

We used the ID converter tool in dictyBase to convert the DDB IDs from the identified proteins to UniProt Accession Numbers (ACs). The UniProt ACs were then entered in PANTHER Gene List Analysis to obtain a functional classification. Both the UniProt ACs and the information from PANTHER were added to the excel files. We then obtained the UniProt KB (UniProt IDs). The UniProt ACs were entered in DAVID to perform a functional annotation clustering. We repeated the above protocol but changing the first GOTERM parameters to include all GO terms as well.

### 2.3. Multiple Alignment of Proteins with Unknown Function

In order to retrieve and align the amino acid sequences of the proteins with unknown functions we used the alignment UniProt tool (https://www.uniprot.org/align/). UniProt identifiers were used. Up and downregulated proteins were run in separate windows.

### 2.4. Transcriptomic Profiling Analysis in Response to cAMP in Dictyostelium

In order to find novel early developmentally regulated proteins our proteomic data was compared to the previous transcriptomic assay mentioned above [39]. Briefly, the previous transcriptomic assay included *D. discoideum* wild-type AX4 strain gene expression (mRNA) profiles, from two experimental time courses: Development on filters and cAMP stimulation in suspension [55]. Despite AX2 and AX4 strains have some genomic and behavioural differences (dictyBase.org) and there are likely to be strain differences under the experimental conditions analyzed in this study, we argue that any changes shared between the two strains provide with robust data. Out of the two cAMP-stimulating conditions, development on filters and in suspension, the suspension samples were used for the purposes of our analysis, since they were done under similar experimental conditions as our proteomic assay (cAMP-stimulation in suspension). The transcriptomic dataset is composed by 29 samples at different timepoints, from which the normalized RNASeq data was used, that according to the authors, was scaled using mappable exon lengths (similar to RPKM, but using uniquely mappable parts of the exon instead) [55]. The differential expression analysis of the data was also done using limma [56].

In a first analysis, gene expression data from cAMP-pulsed AX4 cells in suspension at time point 0 h was tested against gene expression at time point 5 h; we selected the 5 h window since it represents the most similar experimental condition to our proteomic assay, in which cells were stimulated with cAMP for 5 h 30 min. Additionally, as a validation of these observations, we analyzed RNA-Seq transcriptomic data between 0 h and 6 h cAMP stimulated cells, obtaining similar results to the ones found at time point 5 h.

## 3. Results and Discussion

### 3.1. Identification of Protein Level Changes in Response to cAMP Pulsing in Dictyostelium Discoideum

**Figure 1:**
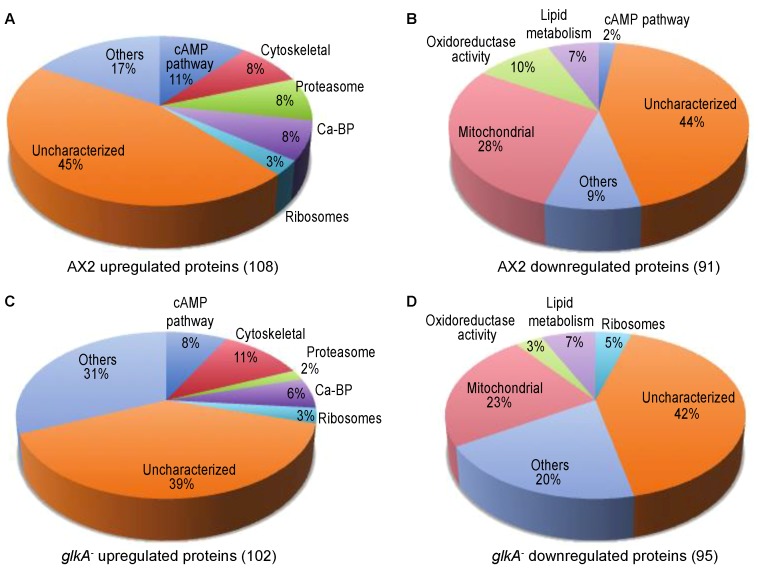
Charts showing differential protein level changes in developed (cAMP-pulsed) versus vegetative (non cAMP-pulsed) wild-type AX2 and *glkA* null (*glkA*^−^) cells. Proteins were further classified based on their biological function. (**A**) 108 upregulated proteins in AX2 cells. (**B**) 91 downregulated proteins in AX2 cells. (**C**) 102 upregulated proteins in *glkA*^−^ cells. (**D**) 95 downregulated proteins in *glkA*^−^ cells.

Out of a total of 5149 proteins in the MS/MS data set, our analyses identified 199 proteins with statistically significant level changes in developed cells compared to vegetative wild-type AX2 cells. 108 proteins were upregulated (Figure 1A, Table S1) whereas 91 were downregulated (Figure 1B, Table S2). Under the same comparison, developmental versus vegetative, a total of 197 significant protein level changes were detected in *glkA* null cells, from which 102 proteins were upregulated (Figure 1C, Table S3) and 95 downregulated (Figure 1D, Table S4). Interestingly, 57 (29 upregulated and 28 downregulated) proteins overlapped in AX2 and *glkA* null cell’s proteomes (Table 1; Table S5). Our results provide with statistically significant bona fide proteins that change in level during the first five h of development in the presence of exogenous cAMP pulses.

### 3.2. The Comparison of Proteomics and Transcriptomics Data Yielded a Significant Number of Concordant Genes

When we analyzed the expression level of the genes coding for the 108 proteins that we found upregulated using proteomics in developed AX2 cells, 65 of them were also found significantly (limma FDR *p*-value < 0.05) upregulated in transcriptomics (0 h versus 5 h contrast) (Table S6, Figure S1). Indeed, there is a 60.2% of concordance between proteomics and transcriptomics, *p*-value = 0.021 exact binomial test, yielding a strong protein-coding gene signature of early developmentally regulated candidates. Additionally, out of the 91 proteins identified as downregulated using proteomics in developed AX2 cells, 71 of their coding genes were also significantly downregulated (limma FDR *p*-value < 0.05) in transcriptomics (0 h versus 5 h contrast). Our analysis shows a 78% of concordance between downregulated candidates in proteomics and transcriptomics, *p*-value = 3.62 × 10^−8^ exact binomial test of the intersection (Table S7, Figure S1). As expected, when we analyzed RNA-Seq transcriptomic data between 0 h and 6 h cAMP stimulated cells, since our proteomics data used cells collected after 5 h 30 min of pulsing in suspension, the results obtained were similar to the ones found at time point 5 h (Figure S1).

### 3.3. Proteins of the Canonical cAMP-Pathway, the Ubiquitin-Proteasome Pathway, Calcium Binding, and Cell Adhesion are Among the Most Abundant c-AMP-Responsive Proteins Upregulated in Early Development

**Figure 2:**
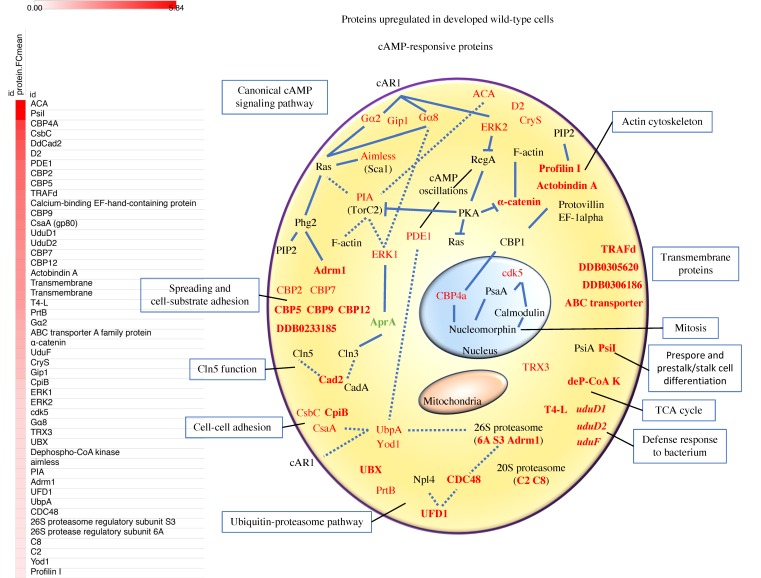
Overview of the cell structure and function. The diagram illustrates the in situ flow of upregulated proteins (mark in red) in early differentiation as a consequence of cAMP stimulation. Novel early developmentally regulated proteins are in bold. Blue lines indicate direct interactions whereas dotted lines indicate they are part of the same signaling pathway or biological process.

The comparison of proteomics and transcriptomics yielded 65 early developmentally regulated proteins upregulated in early development. Out of these, our results include 22 proteins previously annotated as cAMP-responsive proteins, including but not limited to proteins of the canonical cAMP-signaling pathway (Figure 2). On the other hand, our analysis allowed us to identify 43 early developmentally regulated proteins. We have analyzed all of them individually and assigned them with a biological role based on database annotations, orthologs information, and previous published findings.

#### 3.3.1. cAMP-Signaling Pathway

Our results show and validate proteins previously associated with the canonical cAMP-signaling pathway including but not limited to Gα2, Gα8, ACA, phosphodiesterase PDE1, ERK1, and ERK2. When cAMP binds to the surface receptor cAR1, the activity of ACA is stimulated more than five-fold, which stimulates the dissociation of the trimeric G protein subunits, Gα2 and Gβ/Gγ [57]. ACA signal increases more than 40-fold during the first 6 h of development, to generate the cAMP used as a chemoattractant, and is responsible for most of the adenylyl cyclase activity during aggregation [40,58]. Aimless, a GEF factor for RasC [59], is required for ACA activation [60] and is a member of the Ras signaling complex, termed Sca1 complex. The Sca1 complex regulates the RasC-TorC2-Akt/PKB pathway at the leading edge of chemotaxing cells, and undergoes TorC2 and Akt/PKB-dependent negative feedback regulation [61].

Protein kinase ERK1 is a member of the MAP kinase family, involved in polyphosphate signal transduction [62] and in the signal transduction pathway responsible for AprA-dependent chemorepulsion through Gα8 and also TorC2 [63]. On the other hand, ERK2 is a MAP kinase that is essential for aggregation and cAMP relay [64], and it has a central role in regulating chemotaxis [65]. ERK2 is activated by cAMP binding to cAR1 [66] and reversibly inhibits RegA, the major intracellular cAMP phosphodiesterase, which reduces the turnover of internal cAMP such that it can accumulate and activate PKA.

A feedback loop, in which PKA activity inhibits ERK2 inhibition of RegA, leads to an increase in cAMP phosphodiesterase activity that reduces the concentration of cAMP. As a result of this circuit, and the activation of ACA when cAMP binds to cAR1, cAMP oscillates with a period of about 6 min during early aggregation [67,68]. PDE1, which is the major extracellular cAMP phosphodiesterase, is also part of this circuit [67,68,69], and it acts in the extracellular environment to reduce cAMP in the back of the cells and resets the oscillator [70]. Pianissimo (PIA) is also required for chemotaxis and positive regulation of ACA activity [71]. PIA is involved in the signal transduction pathway responsible for AprA-dependent chemorepulsion [63]. Last but not least, cAR1 activation induced G-protein translocation from the cytosol to the plasma membrane is dependent on Gip1, a PH domain-containing protein that offers an explanation for the ability of chemotactic cells to recognize chemical gradients over a wide range of concentrations [72]. D2 and CryS were also previously related to the cAMP-signaling pathway. In particular, D2 was shown to be necessary for proper chemotaxis [73].

#### 3.3.2. Calcium-Binding Proteins (CBPs)

Calcium ions are involved in the regulation of diverse cellular processes such as chemotaxis, cell adhesion, and multicellular development through their interactions with CBPs [74,75,76]. Among the sixty proteins containing a calcium-binding domain in *Dictyostelium*, we have identified four of them as early developmentally regulated proteins, including DDB0233185, CBP5, CBP9, and CBP12. We also found CBP4a upregulated, in contrast to previous observations [77]. CBP4a is a nucleolar protein that interacts with CBP1, important for normal cell aggregation by regulating the actin cytoskeleton [78,79]. CBP4a also interacts with nucleomorphin, which is a cell cycle checkpoint protein, in a Ca^2+^-dependent manner, reason why CBP4a was suggested to function during mitosis [80,81,82]. Interestingly, nucleomorphin also interacts with PsaA and calmodulin, and these two also interact with the cyclin-dependent kinase 5 (highly similar to mammalian cdk5). *Dictyostelium* PsaA is similar to PSA from *Drosophila*, mouse, and human, where it is associated with cell cycle progression and several human diseases including Huntington’s and Alzheimer’s disease [83]. Our results show that Cdk5 is upregulated in developed cells. Cdk5 is a serine/threonine kinase that phosphorylates the cAMP/PKA pathway, which plays a major role in physiology, such as the control of metabolism, apoptosis, and cell differentiation. Interestingly, this pathway induces fatty acid utilization and energy expenditure in peripheral tissues in higher eukaryotes [84,85,86]. In addition, both Cdk5 and cAMP/PKA regulate fundamental central nervous system (CNS) functions including neuronal survival, neurite and axonal outgrowth, neuron development and cognition [87]. CBP9, despite containing three EF-hand motifs, does not bind Ca^2+^ [88], whereas the other three CBPs identified in this study, DDB0233185, CBP5, and CBP12, contain canonical EF-hand motifs that mediate their Ca^2+^-binding properties [89]. The other two previously studied CBPs we find upregulated in this study and that validate previous findings are CBP2 and CBP7. CBP7 plays an important role in cell spreading and cell-substrate adhesion [90], whereas CBP2 mRNA concentration was shown to peak during cellular aggregation [91]. For all these CBPs, their exact functions remain unknown.

#### 3.3.3. Adhesion Proteins and Cln5 Function

It has been shown that the rate of Ca^2+^ influx was stimulated by the chemoattractant cAMP, and that the intracellular calcium ions affected cell-cell adhesion and cell fate determination [92,93]. In this context, our results identify a new early developmentally regulated protein, Cad2, which is a putative adhesion molecule similar to CadA, a calcium-binding and cell-cell adhesion protein. Since CadA (and also AprA) is linked to Cln3 function in *Dictyostelium* [94], we suggest that Cad2 may be linked to Cln3 function as well, and therefore interact with Cln5, a protein necessary for adhesion and chemotaxis [95]. Since Cln proteins in humans are involved in neuronal ceroid lipofuscinoses (NCLs), a group of devastating neurological disorders, we encourage to continue the use of *Dictyostelium* as a biomedical model to study the cellular roles of NCL proteins as recently reviewed [94].

Our analysis also identified Adrm1 which interacts with the tyrosine Phg2 kinase in a pathway that plays a role in the transition from growth to differentiation [96]. Adrm1 is an ortholog of the mammalian adhesion-regulating molecule ADRM1, a component of the 26S proteasome, where it acts as a ubiquitin receptor and recruits the deubiquitinating enzyme, ubiquitin carboxyl-terminal hydrolase L5 [97]. Increased levels of the encoded protein are associated with increased cell adhesion, which is likely an indirect effect of this intracellular protein, whereas dysregulation of this gene has been implicated in carcinogenesis [98]. Phg2 regulates cell substrate adhesion, phagocytosis, motility, and actin organization, in part, by its ability to bind to Adrm1, Rap1, the ras binding domain of RasG and RasS, and its interaction with PIP2 [96].

Our results show that the cell surface glycoproteins CsaA and CsbC are early developmentally regulated proteins, as previously suggested by their expression after 6 h of development when the cells begin to aggregate [99,100,101]. CsaA mediates cell-cell adhesion responsible for contact sites A, whereas the contact site B protein CsbC has not been further characterized. We suggest that both contact site A and B proteins have overlapping functions and, as are Phg2 and Adrm1, are involved in the transition from vegetative to developmental stages in *Dictyostelium*.

#### 3.3.4. Actin Cytoskeleton

Our results have identified actobindin A and profilin I as early developmentally regulated proteins. Actobindins are small proteins with two actin-binding WH2 domains, and very similar to *Acanthamoeba castellanii* actobindin. In this organism, actobindin was shown to bind to two actin monomers at high concentrations of G-actin [102]. Profilins also bind actin as well as PIP2 [103]. Profilin I shows up during early development, its mRNA abundance peaks at 11 h of development [55] and is involved in F-actin regulation, cytokinesis, and development. Another protein that we have identified is α-catenin, which associates with F-actin [104] and for which mutant analysis showed its involvement in adhesion dependent cytokinesis [105]. Furthermore, α-catenin is essential for apical myosin localization during epithelial tube morphogenesis, by regulating cortexillin and Ras-GTPase distribution [104], and for cell-cell adhesion and contact-dependent growth inhibition [106]. Its human ortholog is vinculin, which is involved in cell-matrix adhesion and cell-cell adhesion, playing important roles in cell morphology and locomotion [107].

#### 3.3.5. The Ubiquitin-Proteasome Pathway

The *Dictyostelium* genome harbors several ubiquitin, ubiquitin ligases and around 50 putative deubiquitinating enzymes (DUBs) encoding genes [108]. These proteins can either antagonize or facilitate substrate presentation to the proteasome, setting the proper expression level of specific regulatory factors, controlling progression between stages of development. Indeed, one role of DUBs is the regulation of protein degradation by the 26S proteasome, in this context, our results have identified UFD1 as an early developmentally regulated protein. UFD1 is a ubiquitin fusion degradation protein, similar to *S. cerevisiase* and human UDF1, that together with Npl4 and CDC48 (also identified in this study), is involved in the recognition of polyubiquitinated proteins and their presentation to the 26S proteasome for degradation [109]. Our results also identify as early developmentally regulated proteins the 6A and S3 26S proteasome subunits, as well as the C2 and C8 subunits of the 20S proteasome. None of these proteins, but CDC48, have been previously annotated as early developmentally regulated proteins in *Dictyostelium*. However, recent data suggest that cAMP rapidly enhance 26S proteasome activity in several types of human cells [110]. We suggest that proteasomal early developmentally regulated proteins are important in the turnover of proteins involved in early aggregation. Our results further validate previous findings, including the cAMP-responsive protein PrtB, which is annotated as an orthologue of proteosomal α-subunit 7-1 [111].

Our results identify UBX which contains a ubiquitin interacting motif and it is similar to yeast UBX5 and human UBX7 proteins. In human cells, UBX7 causes the accumulation of the CUL2 substrate HIF1α, which may negatively regulate the ubiquitin-ligase activity of CRL2, preventing recruitment of ubiquitin-receptors other than p97 to nuclear HIF1α [112]. Our study is the first one relating *Dictyostelium* UBX7 and cAMP signaling. On the other hand, the deubiquitinating enzymes found in our analysis, UbpA and Yod1, were previously reported to be important for cAMP wave formation and aggregation [113,114]. We have also identified CpiB, which is one of the three homologs of mammalian cysteine protease inhibitors, and capable of inhibiting cysteine proteases in vitro [115].

#### 3.3.6. Proteins with Unknown Functions

We have identified three proteins previously shown to be upregulated in the uninfected *dupA* mutant, a key regulator of the amoebal MAP kinase response to *L. pneumophila* [116]. Some of the amoebal genes appear to be involved in a response similar to innate immunity in higher eukaryotes, indicating there was misregulation of a conserved response to bacteria [116]. Indeed, DupA is similar to MAP kinase phosphatase in plants, and dual-specifity phosphatase 19 in mammals. T4-L might be also involved in the defense response to bacterium.

Our results suggest that PsiI is an early developmentally regulated protein which may also have a role during aggregation. PsiI is a PA14 domain-containing protein similar to prespore inducing factor PsiA [117]. During normal development, the *psiA* gene is highly expressed in scattered cells at the mound stage and in prespore cells at the onset of culmination, and regulates both prespore and prestalk/stalk cell differentiation [118]. Last but not least, we have identified four transmembrane proteins, one metabolic protein, two LIM-type zinc finger-containing proteins (DDB0233465 and DDB0238324) and 15 unique proteins with unknown functions that have not been previously annotated or studied (Table S6). Our protein sequence-based analysis showed that all these 15 protein sequences are unique, with no domains or regions in common.

### 3.4. Mitochondrial Proteins Related to Metabolism, Proteins Involved in Fatty Acid, Coenzyme A, and Steroids Synthesis are Among the Most Abundant Proteins Downregulated in Early Development

**Figure 3:**
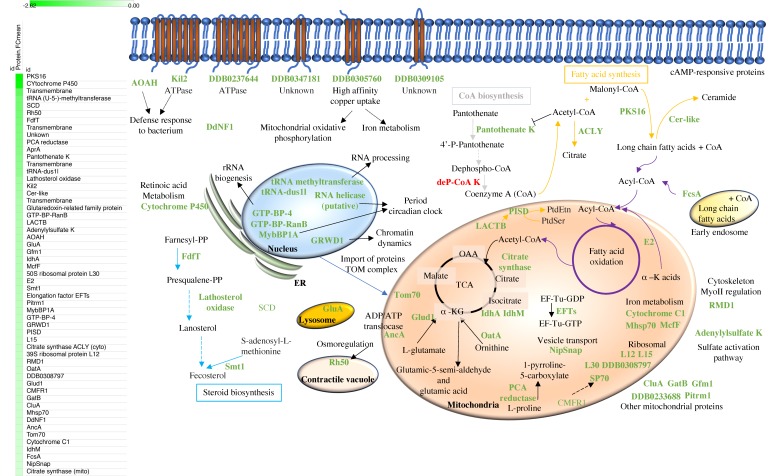
Diagram that illustrates the in situ flow of downregulated proteins (mark in green) in early development as a consequence of cAMP stimulation. Novel early developmentally regulated proteins are in bold. Arrows indicate direct interactions or contiguous proteins in a signaling/enzymatic cascade, whereas dotted arrows indicate they are part of the same signaling pathway or biological process but not contiguously.

Our proteomic and transcriptomic analyses show an early developmentally regulated signature of 71 downregulated proteins in developed versus vegetative cells. Out of these, four proteins were previously annotated by others as cAMP-responsive proteins, whereas 67 have been identified in this study as early developmentally regulated proteins for the first time (Figure 3). As we did above, we have analyzed all these proteins individually and assigned them with a biological role based on their biological annotations, orthologs information, and previous published findings.

#### 3.4.1. Fatty Acid Metabolism

Fatty acids have four major physiological roles: They are the building blocks of phospholipids and glycolipids, many proteins are modified by the covalent attachment of fatty acids, which targets them to membrane locations, are oxidized to meet the energy needs of a cell or organism, and their derivatives serve as hormones and intracellular messengers. Among the proteins that exert these functions, *D. discoideum* is exceptionally rich in polyketide synthases (PKS), a large family of multifunctional proteins involved in the biosynthesis of diverse classes of natural products [119,120]. In this study we have identified PKS16, a β-ketoacyl synthase family protein that catalyzes the formation of long-chain fatty acids from acetyl-CoA, malonyl-CoA, and NADPH. Its human ortholog is known as FASN. A different type of enzyme, fatty acyl-CoA synthetase A (FcsA), catalyze the formation of thioester bond between a free long fatty acid and coenzyme A, playing a role in the retrieval of fatty acids from the lumen of phagosomes into the cytosol [121]. ACLY or ATP-citrate synthase, is the primary enzyme responsible for the synthesis of cytosolic acetyl-CoA in many tissues, which has a central role in de novo lipid synthesis. In nervous tissue, it may be involved in the biosynthesis of acetylcholine. Cer-like is an acid ceramidase-like protein, weakly similar to acid ceramidase, that has not been characterized in *Dictyostelium*.

SCD is an endoplasmic reticulum (ER) stearoyl-CoA-desaturase, a key enzyme in fatty acid metabolism. It contains a cytochrome b5 domain that introduces a double bond at the delta position of fatty acids during the biosynthesis of monounsaturated fatty acids. Specifically forms oleate and palmitoleate from stearoyl-CoA and palmitoyl-CoA. Oleate and palmitoleate are major components of membrane phospholipids, cholesterol esters, and alkyl-diacylglycerol, having an impact on membrane fluidity. In humans, SCD1 function was shown to be involved in germ cell determination, adipose tissue specification, liver cell differentiation, cardiac development, as well as in hypertriglyceridemia, atherosclerosis, and diabetes when the protein is overexpressed [122]. Treatment of preadipocytes with cAMP-elevating agents caused an increase in *scd1* mRNA concentrations, maximally after 6 h of cAMP treatment during late adipocyte development in 3T3-L1 cells [123]. Our results suggest that cAMP regulates fatty acid synthesis, with and inhibitory role in early differentiation. Indeed, it was shown that the PKS SteelyA has a regulatory function on cAMP signaling during aggregation and is indispensable for full activation of ACA [120].

#### 3.4.2. Coenzyme A (CoA) Biosynthesis

Coenzyme A functions as a carrier of acetyl and acyl groups and is essential for numerous biosynthetic, energy-yielding, and degradative metabolic pathways. Acetyl-CoA is the common cellular currency for acetyl transfers, notable for its role in the synthesis and oxidation of fatty acids, and the oxidation in the citric acid (TCA) cycle. CoA and its thioester derivatives (acetyl-CoA, malonyl-CoA, etc.) participate in diverse anabolic and catabolic pathways, allosteric regulatory interactions, and the regulation of gene expression [124]. The biosynthesis of CoA requires pantothenate, cysteine and ATP, and involves five enzymatic steps that are highly conserved from prokaryotes to eukaryotes. The intracellular levels of CoA and its derivatives change in response to extracellular stimuli, stresses and metabolites, and in human pathologies, such as cancer, metabolic disorders and neurodegeneration. Indeed, it was recently proposed that cAMP directly binds to *Salmonella enterica* acetyl-coenzymeA synthetase, and inhibits its activity in a substrate-competitive manner [125]. Interestingly, the cAMP contact residues are well conserved from prokaryotes to eukaryotes, suggesting a general regulatory mechanism of cAMP on Acs [125]. Related to the biosynthesis of coenzyme A we have identified the enzymes pantothenate K, and dephospho-coenzyme A kinase (this one was upregulated). Pantothenate K phosphorylates pantothenate to 4’-phosphopantothenate in the first step of the pathway, whereas dephospho-coenzyme A kinase acts in the last step. Therefore, pantothenate K plays a role in the physiological regulation of the intracellular CoA concentration. This enzyme is strongly inhibited by acetyl-CoA and by manyl-CoA, and also inhibited by high concentration of non-esterified CoA (CoASH).

#### 3.4.3. Steroid Biosynthesis

Steroids have two principal biological functions: As important components of cell membranes, which alter membrane fluidity, and as signaling molecules. The cAMP signal transduction pathway is the major signaling cascade in the regulation of steroidogenesis. This regulation is principally dictated by the StAR protein, a rapidly synthesized mitochondrial phosphoprotein, influenced by both cAMP/PKA-dependent and cAMP/PKA-independent signaling pathways [126]. In *Dictyostelium*, steroids initiate a signaling cascade that triggers rapid sporulation [127]. In this work we have identified FdfT, lathosterol oxidase and Smt1 downregulated in response to cAMP. All three proteins are involved in steroid biosynthesis, and they operate in the ER, although they have not been previously characterized in *Dictyostelium*. FdfT produces an intermediate in both the mevalonate and non-mevalonate pathways, used by organisms in the biosynthesis of terpenes, terpenoids, and sterols as well as it is used in the synthesis of CoQ. Lathosterol oxidase catalyzes a dehydrogenation to introduce C5-6 double bond into lathosterol. Disfunction of this protein cause lathosterolosis, an autosomal recessive disorder characterized by a complex phenotype, including multiple congenital anomalies, mental retardation, and liver disease. Smt1 catalyzes the methyl transfer from S-adenosyl-methionine to the C-24 of zymosterol, to form fecosterol. The information provided here is based on their human orthologs.

#### 3.4.4. RNA Processing

Our results identify a putative RNA helicase and two tRNA processing proteins, all three conserved in higher eukaryotes. The RNA helicase is similar to human DDX5 and DDX7, which are RNA helicases involved in alternative regulation of splicing. As a component of a large PER complex, it is involved in the inhibition of 3’ transcriptional termination of circadian target genes, such as the period circadian clock (Per) PER1 and NR1D1. The tRNA synthase dus1l is an ortholog of *S. cerevisiae* DUS1, and the animal DUS1L; catalyzes the synthesis of dihydrouridine, a modified base found in the D-loop of tRNAs. The other tRNA processing protein is a tRNA (uracil-5-)-methyltransferase, that catalyzes the formation of 5-methyl-uridine at position 54 in all tRNA. May also have a role in tRNA stabilization or maturation [128].

In the nucleus of the cells we have also identified GRWD1, and three proteins related to ribosomal biogenesis, MybBP1A, and two GTP binding proteins. GRWD1 is a histone binding-protein that regulates chromatin dynamics and minichromosome maintenance loading at replication origins, possibly by promoting chromatin openness. Interacts with METTL18, CDT1 and with CDC6, and it binds to histone H2A-H2B, and H3-H4 complexes [129,130]. MybBP1A is similar to the human Myb-binding protein 1A, which act as a corepressor and in concert with CRY1 to represses the transcription of the core circadian clock component PER2. Preferentially binds to dimethylated histone H3 ’Lys-9’ (H3K9me2) on the PER2 promoter. It also has a role in ribosomal biogenesis, together with PWP1. The two GTP-binding proteins are GTP-BP4 and GTP-BP-RanB. GTP-BP4 is very similar to NOG1/GTPBP4 GTPases, which in *S. cerevisiae* associate with free 60S ribosomal subunits in the nucleolus, and is involved in their biogenesis. GTP-BP-RanB contains a Ran domain, which is an evolutionary conserved member of the Ras superfamily, that regulates all receptor-mediated transport between the nucleus and the cytoplasm [131]. All the above annotations are by similarity with their human counterparts.

#### 3.4.5. Mitochondrial Proteins

The largest group of early developmentally downregulated proteins identified in this study correspond to mitochondrial proteins. In particular, some of them are part of or related to the TCA cycle, including the mitochondrial citrate synthase, the isocitrate dehydrogenases IdhA and IdhM, OatA and Glud1. Citrate synthase catalyzes the condensation of mitochondrial acetyl-CoA and oxaloacetate to citrate. IdhA is the *Dictyostelium* ortholog of the α subunit of mitochondrial NAD+-dependent isocitrate dehydrogenase NAD+, which catalyzes the oxidative decarboxylation of isocitrate into α-ketoglutarate, the third step in the TCA. IdhM is a NADP+-dependent isocitrate dehydrogenase. Ornithine aminotransferase OatA catalyzes the transfer of an amino group from ornithine to α-ketoglutarate, yielding glutamic-5-semi-aldehyde and glutamic acid. GluD1, produces α-ketoglutarate from L-glutamate; acting on the CH-NH2 group of donors with NAD+ or NADP+ as acceptor. Related to fatty acid oxidation we have identified E2, a mitochondrial component of branched-chain keto acid dehydrogenase, that transfers 2-methylpropanoyl, 3-methylbutanoyl, or S-2-methylbutanoyl groups to coenzyme A.

LACTB is a mitochondrial serine protease that acts by decreasing protein levels of PISD, a mitochondrial enzyme that converts phosphatidylserine (PtdSer) to phosphatidylethanolamine (PtdEtn), thereby affecting mitochondrial lipid metabolism [132]. In humans, LACTB acts as a tumor suppressor, that has the ability to inhibit proliferation of multiple types of breast cancer cells [132]. *Dictyostelium* PISD is very similar to the *H. sapiens* PISD, a mitochondrial proenzyme that is cleaved into a phosphatidylserine decarboxylase α and β chain; contains one putative transmembrane domain where it catalyzes the formation of PtdEtn from phosphatidylserine PtdSer. Plays a central role in phospholipid metabolism, and in the inter-organelle trafficking of phosphatidylserine. Interestingly, phosphatidylethanolamine and phosphatidylcholine metabolism have a critical role in health and disease, in part because they are the most abundant phospholipids in all mammalian cell membranes [133].

Other proteins identified in this study, but whose function is less characterized, include the mitochondrial ribosomal proteins 39S subunits L12 and L15, as well as 50S subunit L30. Cytochrome C1, an ortholog of the heme-containing component of the cytochrome b-c1 complex (CYC1), which accepts electrons from Rieske protein, and transfers electrons to cytochrome c in the mitochondrial respiratory chain. McfF, which is an ortholog of *H. sapiens* SLC25A28 and *D. reiro* SLC25A37, involved in iron transport across the mitochondrial membrane. Mhsp70, a chaperone that plays an important role in mitochondrial iron-sulfur cluster (ISC) biogenesis [134]. The elongation factor EFTs, that associates with the EF-Tu-GDP complex, and induces the exchange of GDP to GTP. In humans its involved in combined oxidative phosphorylation deficiency 3 (COXPD3), a mitochondrial disease resulting in severe metabolic acidosis, with encephalomyopathy or with hypertrophic cardiomyopathy. NipSnap is a mitochondrial conserved protein that may have a role in vesicle transport, whereas Tom70, ortholog of human mitochondrial import receptor subunit TOM34 (component of the TOM complex), might be responsible for import of nuclear-encoded proteins into the mitochondria. Mitochondrial AncA is an ortholog of human SLC25A4, which catalyzes the exchange of ADP and ATP across the mitochondrial inner membrane; contains six predicted transmembrane domains. Mutations affecting this protein cause progressive external ophthalmoplegia autosomal dominant disease. PCA reductase, transform L-proline into 1-pyrroline-5-carboxylate. Its human ortholog (PYCR1) catalyzes the last step in proline biosynthesis, and it is involved in the cellular response to oxidative stress.

Last but not least, we have also identified the mitochondrial proteins GatB and Gfm1, which are involved in translation, Pitrm1, and DDB0233688, related to protein degradation, and CluA, involved in cytokinesis. GatB is a subunit of the mitochondrial glutamyl-tRNA amidotransferase, whereas Gfm1 is involved in the translocation of the nascent peptide from the ribosomal A-site to the P-site, suggesting a reduction in the speed of translation at the mitochondrial level. Pitrm1 is a pitrilysin metalloprotease, ortholog of *H. sapiens* PITRM1 and *S. cerevisiae* CYM1, involved in protein degradation. CluA is responsible for distribution of mitochondria in the cell and cytokinesis. Cytosolic RMD1, regulator of microtubule dynamics protein, is required for myosin II cleavage furrow accumulation and plays a role in cell separation during cytokinesis [135]. Our results show that both CMFR1 and its downstream effector SP70 are downregulated in early differentiation. CMFR1 is a putative receptor mediating cell-density sensing which function is essential for proper aggregation [136] and it also induces SP70, which accumulates in a subset of prespore cells.

#### 3.4.6. Defense Response to Bacterium

We have identified two proteins related to defense response to bacterium, Kil2, and AOAH. P-type ATPase Kil2 is a transmembrane magnesium pump, involved in maintaining the phagosomal magnesium concentration, optimal for proteolysis and efficient killing of bacteria. Its deficiency causes an aberrant defense response to bacterium [137]. AOAH, ortholog to human acyloxyacyl hydrolase removes the secondary fatty acyl chains from the lipid A region of bacterial lipopolysaccharides [138].

*Β-glucosidase* was one of the first developmentally regulated enzymes to be used as a stage specific marker in *Dictyostelium* [139], and our data corroborate previous mRNA levels for the beta-glucosidase GluA [55,140]. This protein is found in lysosomes and secreted into the surrounding medium.

#### 3.4.7. Uncharacterized Proteins

This section contains those proteins identified in our study that are downregulated and whose function in *Dictyostelium* has not been characterized at all. The information summarized here correspond to data obtained from their orthologs in higher eukaryotes. Cytochrome P450 family protein CYP524A1 is a membrane protein of the ER, that in human plays a central role in germ cell development, involved in the metabolism of retinoic acid. Rh50, similar to human Rh50 protein, a transmembrane glycoprotein present on the surface of red blood cells [141]; its human ortholog, when mutated, is involved in several human diseases (Rh-null hemolytic anemia and overhydrated hereditary stomatocytosis). DDB0305760, is similar to *H. sapiens* solute carrier family 31 member 1 (SLC31A1) or high affinity copper uptake protein 1; contains three predicted transmembrane regions. DDB_G0277011 is highly similar in an N-terminal domain to the Shaker voltage-gated potassium channel of vertebrates, and is the best hit in the *Dictyostelium* genome when searched with the sequence of this channel. The channel is strongly expressed in several classes of neurons in adult animals, and appears to regulate neuronal excitability. The channel is also expressed during embryonic development, where it shows a correlation with proliferation [141]. Previous work shows clear correlation between expression and proliferation in cultured Schwann cells, and blockade of potassium conduction inhibits proliferation (reviewed in [142]). Glutaredoxin-related family protein (DDB0233688) is related to glutaredoxin, which functions as an electron carrier in the glutathione-dependent synthesis of deoxyribonucleotides. DDB0349243 is an ortholog of human Cathepsin B, which is believed to participate in intracellular degradation and turnover of proteins, including the matrix extracellular phosphoglycoprotein MEPE [143]. It has also been implicated in tumor invasion and metastasis [144]. Adenylylsulfate kinase has a human ortholog named PAPSS2, which is a bifunctional enzyme with both ATP sulfurylase and APS kinase activity, that mediates two steps in the sulfate activation pathway.

#### 3.4.8. Unknown Proteins

In the case of downregulated proteins, we have identified 16 proteins with unknown functions, that we annotate as early developmentally regulated proteins. Our protein sequence-based analysis showed that all these sequences are unique, with no domains or regions in common.

### 3.5. Proteomics in glkA-null Cells

As we mentioned above, using whole-cell proteome analysis of glkA null cells we identified 197 protein level changes between developed (cAMP-pulsed) and vegetative (non cAMP-pulsed) cells; 102 proteins were upregulated whereas 95 were downregulated (Figure 1C,D, respectively). The group of upregulated proteins include but it is not limited to 41 uncharacterized proteins, 11 cytoskeletal proteins such as acting proteins, eight related to the cAMP-pathway, and six calcium-binding proteins (Figure 1C; Table S3). This functional annotation was corroborated with the different annotation clusters obtained with DAVID analysis (Table S3). Out of the eight proteins related to the cAMP-pathway, five of them were also found upregulated in AX2 cells including the cAMP receptor CAR1, ERK2, the cyclin-dependent kinase 5 homolog, Gα-2 and LIME (Table 1). The other three proteins are Rac1B, PAKa, and Ca-binding protein A (CBP1). Rac1B regulates the basal levels of F-actin assembly, its dynamic reorganization in response to chemoattractans and cellular polarity during chemotaxis [145]. PAKa is involved in the regulation of the cytoskeleton during chemotaxis as a major regulator of myosin II assembly and is regulated by the PI3K/Akt pathway [146,147]. CBP1 also regulates reorganization of the actin cytoskeleton during cell aggregation [78]. Other proteins upregulated in glkA null cells and AX2 cells included but are not limited to actobinding-A, cystatin A2, 3 Ca-binding proteins, and 15 uncharacterized proteins.

Apart from the upregulated proteins, our analysis identified 95 proteins downregulated in developed glkA null cells. This group includes but it is not limited to 46 uncharacterized proteins (including seven putative mitochondrial proteins, two putative proteins involved in lipid metabolism, and one putative ribosomal protein), 20 mitochondrial proteins, six involved in lipid metabolism, five transporters and four thiol proteases (Table S4). This functional annotation was corroborated with the different annotation clusters obtained with DAVID analysis (Table S4). Twenty-eight out of these 95 proteins were also found downregulated in AX2 cells. Among these proteins, the biggest group based on GO corresponded to 13 uncharacterized proteins, followed by proteins involved in metabolic processes and lipid metabolism (Table 1).

### 3.6. Identification of GlkA-Dependent Candidates

Since wild-type AX2 and *glkA* null cell’s proteomics samples were run in different 4-iPLEX in the MS/MS, rather than doing a direct quantitative comparison we decided to compare both proteomes from a qualitative point of view. Our proteomics analysis identified up to 26 proteins in *glkA* null cells that were not detected at all in AX2 cells, suggesting that their coding genes are directly silenced by GlkA (Table S8). Eleven out of these 26 proteins are uncharacterized, and the most abundant protein with annotated domains is an ABC transporter G family protein. Mutants in this gene have decreased fruiting body size and decreased spore viability [148]. Further functional annotation clustering of these 26 proteins using DAVID annotation tool did not provide any significant (*p* ≤ 0.01, Benjamini Test) hit, the lowest p value for a given cluster was over 0.35, suggesting these proteins are regulating different aspect of the biological response to cAMP signaling.

On the other hand, our proteomics analysis identified 20 proteins in AX2 cells that were not detected at all in *glkA* null cells, suggesting that GlkA is essential for the expression of their corresponding genes (Table S9). Not surprisingly since GlkA effectors are little characterized; 17 of these proteins have unknown functions and have not been previously studied. The other three proteins are the calcium binding protein CBP12, the cAMP/cGMP dependent phosphodiesterase PDE1, and the carboxylesterase D2. Indeed, D2 carboxylesterase is very upregulated in developed AX2 cells, it was found to regulate MyoII, and D2 mutants showed chemotaxis defects [45]. The CBP12 is very similar to *Dictyostelium* CBPF, CBPG, and CBPC, and contains two EF-hands, which are involved in binding intracellular calcium. Although CBP12 has not yet been fully characterized, according to our own proteomics data CBP12 is highly upregulated in developed AX2 cells. PDE1 is the mayor extracellular cAMP phosphodiesterase in *Dictyostelium*, which functions to maintain the responsiveness of cells to the chemoattractant cAMP during the aggregation phase of development [67,68,69]. PDE1 mutants have aberrant chemotaxis and abolished aggregation.

**Figure 4:**
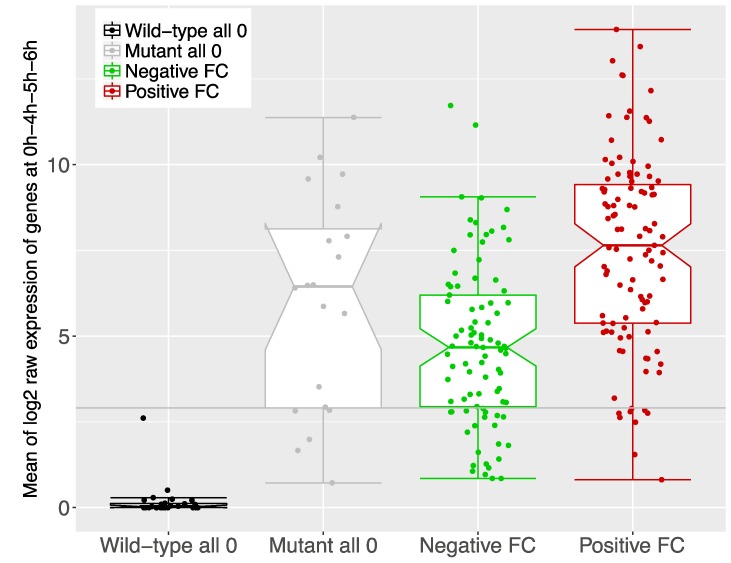
Mean of log2 raw expression of genes at 0 h, 4 h, 5 h, and 6 h based on the global basal mean expression using the transcriptomic data for different groups of proteins identified in proteomics. From left to right, group of coding genes that were identified in the proteome of *glkA* null cells but not in the proteome of wild-type cells (Wild-type all zero); group of coding genes that were identified in the proteome of AX2 cells but not in the proteome of *glkA* null cells (Mutant all zero); group of coding genes found significantly differentially expressed, either downregulated (Negative FC) or upregulated (Positive FC). FC stands for fold-change.

We then examined the transcriptomic signal of all the above 46 protein-coding genes. Our results show how in transcriptomics the raw log2 expression signal corresponding to the group of genes not detected in AX2 (Wild-type all 0 in Figure 4) is significantly below the basal threshold, which matches with our proteomics results. Therefore, these results strongly support that GlkA is downregulating the expression of at least these 26 genes (Figure 4; Table S8. On the other hand, the 20 proteins detected in AX2 cells, but not in *glkA* null cells, were also found in transcriptomics, suggesting their expression is dependent on GlkA (Figure 4; Table S9).

## 4. Conclusions

The identification of cAMP as the chemoattractant in *Dictyostelium* [149], opened up the analysis of cell signaling to biochemical and molecular biological techniques with which it was possible to recognize and characterize the enzymes that synthesize cAMP, the surface receptors for cAMP, and many of the components of the signal transduction pathways [150,151,152,153,154]. These advances solidified the position of *Dictyostelium* as a model organism to study chemotactic motility and multicellular development.

This work identifies protein level changes between the proteome of vegetative (non cAMP-pulsed) and developed (cAMP-pulsed) wild-type AX2 cells based on a proteomics study done under experimental conditions that mimic the signal propagation during aggregation in early development in *Dictyostelium*. Under the same experimental conditions and analyses we also identified protein level changes between the proteome of vegetative and developed *glkA* null cells; GlkA is a protein that was identified as a GSK3 family kinase member that regulates *Dictyostelium* cell’s cytoskeleton via key cAMP signaling proteins [45]. Interestingly, both in AX2 and *glkA* null cells, the largest group identified correspond to proteins of unknown function, which now we propose are related to early development and perhaps to cAMP signaling. These uncharacterized proteins, they all have in common that they do not have annotated domains, never been previously studied and no function has been inferred. Apart from the canonical proteins previously known to have a role in cAMP signaling our data also support the previous notion that Ca^2+^ chemotaxis is co-acquired with cAMP chemotaxis during early development, corroborate the importance of calcium-binding proteins during chemotaxis [155], and provide with new candidates to participate in this process. We also identified cytoskeletal, ribosomal, and proteasomal proteins to be upregulated in developed cells, compare to vegetative cells in early development. All together, these increased protein levels may be necessary to properly orchestrate a fine tune regulation during cell migration. On the contrary, proteins involved in metabolism were rather downregulated, including proteins involved in lipid metabolism, oxidoreductase activity, and mostly mitochondrial related proteins. Our data suggest that the proteome is shutting down the metabolism of the cells, presumably to reduce growth, cell division, and other metabolic characteristics most typical in vegetative cells. Interestingly, the comparison of the proteomics results obtained in AX2 and *glkA* null cells suggest that despite the different changes identified in protein levels between the two strains, the big picture remains closely intertwined. Ca binding proteins, cytoskeletal, proteasomal, and ribosomal proteins are upregulated in developed versus vegetative *glkA* null cells. Whereas proteins related to the metabolism of the cells are downregulated.

Since *glkA* null cells have aberrant chemotaxis defects we thought it would be of great value to incorporate a bioinformatic analysis to identify proteins in wild-type cells that where not present in cells lacking GlkA, and the other way around. Therefore, our analysis aimed at identifying two group of proteins: Proteins that overlap between the two strains despite the absence of GlkA, which provides with robust changes responding to cAMP that are independent of the GlkA kinase, and another group of proteins which are either detected or not under our experimental conditions in proteomics in a GlkA-dependent manner. This analysis has provided potential downstream effectors of GlkA in early development as well as developmentally regulated proteins that are independent of GlkA regulation. This analysis reveals an overlap of 57 proteins in both AX2 and *glkA* null cells, whereas it also shows 46 proteins that show differences in expression between AX2 and *glkA* null strains. In this work, we provide the list of all the identified proteins and their known functions.

Using pre-existing published transcriptomic and proteomic data sets during early *Dictyostelium* development we have identified, using bioinformatics, a large overlap (70%) between both data sets resulting in a signature of 199 early developmentally regulated proteins. Interestingly, 110 are novel proteins that we annotate here as developmentally regulated proteins. This analysis uses two different wild-type *Dictyostelium* strains AX2 for proteomics and AX4 for transcriptomics, albeit in similar developmental conditions, adding weight that the change in levels of these proteins is robust and can be explained at the level of transcription. In addition, there are some that change in one dataset but not the other, perhaps consistent with control at a different level, although the different strains used does make this hard to interpret. It is worth noticing that about 30% of the 199 selected proteins did not experiment the same change in both data sets (transcriptomics and proteomics). This result might be due to particular transcriptional and/or posttranscriptional changes in AX2 (used in proteomics) and AX4 (used in transcriptomics), the detection of the transcripts/proteins, as well as poor statistics or reproducibility of the results generated.

Although the connection between cAMP signaling in *Dictyostelium* and higher eukaryote cells has yet to be proven, we suggest that there is a strong signature of cAMP signaling proteins in *Dictyostelium* that have orthologs in higher eukaryotes, including humans. Moreover, due to the high number of identified proteins that have not been previously characterized and/or have unknown functions, many questions remain to be answered and therefore, further experiments are needed to investigate the role of cAMP in early differentiation and its translation to higher eukaryote cells. The use of *Dictyostelium* cells in cell biology and molecular medicine, in particular as a eukaryotic model for cell motility and several human diseases, may shed light on the complexity of the cAMP signaling network in higher eukaryotes and to fight certain pathological conditions. Our results identified novel early developmentally regulated proteins, which contribute to understand the response that cAMP elicits in early differentiation and cell migration.

## Figures and Tables

**Table 1:** Top 57 proteins whose level changes overlapped in both AX2 and *glkA* null cells. Out of the statistically significant 57 proteins, 29 are upregulated (left panel) and 28 are downregulated (right panel) in developed versus vegetative cells. UniProt IDs and protein names, if available, are shown. Color code group proteins based on their biological classification; in the left panel from top to bottom: cAMP-pathway proteins, cytoskeletal, Ca-binding, uncharacterized and other proteins. In the right panel from top to bottom: Proteins involved in lipid metabolism, mitochondrial (including three uncharacterized: Q54JP5, Q8MP58, and Q54IS1), uncharacterized and other proteins.

Proteins Upregulated in Wild-Type and *glkA* Null Cells		Proteins Downregulated in Wild-Type and *glkA* Null Cells	
UniProt	Protein	UniProt	Protein
P13773	Cyclic AMP receptor 1	Q55DR6	Fatty acyl-CoA synthetase A
Q54QB1	ERK2	Q54N49	Inositol-3-phosphate synthase
P34117	Cyclin-dependent kinase 5 homolog	Q54YA0	Probable ATP-citrate synthase
P16051	G alpha-2	Q54I98	Cycloartenol-C-24-methyltransferase
O60952	LIM domain-containing protein E	Q54DR1	Squalene synthase (SQS)
Q55DU3	Actobindin-A	Q553V1	Citrate synthase
Q65YR7	Cystatin-A2	Q54KB7	Glutamate dehydrogenase
Q1ZXH5	Calcium-binding protein	Q55BI2	Isocitrate dehydrogenase subunit A
P54653	Calcium-binding protein 2	O97470	Substrate carrier family protein ancA
Q54RF4	Calcium-binding protein 4a	Q54JP5	Probable ornithine aminotransferase
P54679	Probable membrane ATPase	Q8MP58	Uncharacterized protein
Q86AA1	Probable T4-type lysozyme 2	Q54IS1	Uncharacterized protein
Q54FS0	Uncharacterized protein	Q55EK2	Probable cytochrome P450 524A1
Q54FV6	Uncharacterized protein	Q869W9	Probable polyketide synthase 16
Q54G71	Uncharacterized protein	Q556T4	Uncharacterized protein
Q54GR0	Uncharacterized protein	Q1ZXN5	Uncharacterized protein
Q54I40	Uncharacterized protein	Q54CD7	Uncharacterized protein
Q54IK3	Uncharacterized protein	Q54J99	Uncharacterized protein
Q54Q34	Uncharacterized protein	Q54R89	Uncharacterized protein
Q54UX5	Uncharacterized protein	Q54T87	Uncharacterized protein
Q54WT5	Uncharacterized protein	Q54WK0	Uncharacterized protein
Q556W6	Uncharacterized protein	Q86KA1	Uncharacterized protein
Q55BQ2	Uncharacterized protein	Q54NS9	Apoptosis-inducing factor homolog A
Q55E22	Uncharacterized protein	Q5XM24	Autocrine proliferation repressor protein A
Q86AC9	Uncharacterized protein	Q9GPS1	Complex III assembly factor LYRM7
Q55DE7	Ataxin-2 homolog	Q55G75	PH domain-containing protein
P90532	Cell division cycle protein 48	Q55BZ5	Protein dcd1A
Q54ST6	Membrane protein subunit	Q54F74	Sulfate adenylyltransferase
Q54I92	Protein psiI		

## Supplementary Materials

The following are available online at https://www.mdpi.com/2073-4409/8/10/1187/s1. Figure S1: Comparison of AX2 proteomics and AX4 transcriptomics data; Table S1: Identification of statistically significant 108 proteins upregulated in developed versus vegetative AX2 cells in proteomics; Table S2: Identification of statistically significant 91 proteins downregulated in developed versus vegetative AX2 cells in proteomics; Table S3: Identification of statistically significant 102 proteins upregulated in developed versus vegetative *glkA* null cells in proteomics; Table S4: Identification of statistically significant 95 proteins downregulated in developed versus vegetative *glkA* null cells in proteomics; Table S5: Identification of statistically significant 57 proteins (29 upregulated and 28 downregulated) that overlapped in AX2 and *glkA* null cell’s proteomes; Table S6: Comparison of AX2 proteomics and AX4 transcriptomics data. Out the 108 proteins that we found upregulated in proteomics, 65 of them were also found significantly (limma FDR *p*-value < 0.05) upregulated in transcriptomics (0 h versus 5 h contrast) and are highlighted in pink; Table S7: Comparison of AX2 proteomics and AX4 transcriptomics data. Out of the 91 proteins identified as downregulated in developed AX2 cells in proteomics, 71 of their coding genes were also significantly downregulated and are highlighted in pink; Table S8: Total ITRAQs data, UniProt IDs, and Panther annotations for all 26 proteins identified upregulated in *glkA* null cells; Table S9: Total ITRAQs data, UniProt IDs, and Panther annotations for all 20 proteins identified downregulated in *glkA* null cells.
